# Efficacy of Prophylactic Magnesium Therapy for the Prevention of Cisplatin-Induced Renal Injury: A Meta-Analysis

**DOI:** 10.3390/jcm15166278

**Published:** 2026-08-13

**Authors:** Motoki Nishida, Kohei Jobu, Tomoaki Ishida, Shumpei Morisawa, Akio Odaira, Takumi Maruyama, Yukihiro Hamada

**Affiliations:** 1 Department of Pharmacy, Kochi Medical School Hospital, Kochi University, Nankoku 783-8505, Japan; jm-m.nishida@kochi-u.ac.jp (M.N.); jm-morisawa.s@kochi-u.ac.jp (S.M.); jm-maruyama.takumi@kochi-u.ac.jp (T.M.); hamada_yukihiro@kochi-u.ac.jp (Y.H.); 2 Graduate School of Integrated Arts and Sciences, Kochi University, Nankoku 783-8505, Japan; 3 Department of Clinical Pharmacy, Graduate School of Pharmaceutical Sciences, Nagoya City University, Nagoya 467-8603, Japan; t-ishida@phar.nagoya-cu.ac.jp; 4 Department of Pharmacy, Tokyo Women’s Medical University Hospital, Tokyo 162-8666, Japan

**Keywords:** cisplatin, magnesium, renal injury, meta-analysis

## Abstract

**Background/Objectives**: Cisplatin is used as treatment for various malignant tumors; nevertheless, this anticancer drug frequently causes side effects such as renal damage and hypomagnesemia. Hypomagnesemia has been suggested to cause renal damage, and preventing hypomagnesemia through magnesium supplementation is necessary to continue treatment while maintaining treatment intensity. Although the usefulness of magnesium supplementation has been previously evaluated, problems with the amount of magnesium supplementation and sample size have been identified. This study aimed to examine the usefulness and amount of magnesium supplementation by conducting a meta-analysis of a large number of studies. **Methods**: This meta-analysis adhered to the Preferred Reporting Items for Systematic Reviews and Meta-Analyses reporting guidelines. Relevant studies published in PubMed, Scopus, Ichushi, and Cochrane Library databases from inception to 31 December 2025 were selected. The searched text combinations were “cisplatin” and “magnesium.” **Results**: Magnesium supplementation showed an inhibitory effect on cisplatin-induced renal injury. The inhibitory effect of magnesium supplementation showed no difference among the three dose groups (<10 mEq, 10–<20 mEq, and ≥20 mEq). **Conclusions**: Magnesium supplementation is useful for preventing cisplatin-induced nephrotoxicity, irrespective of its dose. The results of this meta-analysis may be clinically helpful in preventing hypomagnesemia-induced nephrotoxicity and continuing treatment.

## 1. Introduction

Cisplatin is an anticancer drug that exerts antitumor effects by forming cross-links within and between DNA strands. Although cisplatin is considered a key drug in the treatment of lung, gastric, and other cancers, its use is accompanied by serious side effects such as renal damage (cisplatin-induced nephrotoxicity [CIN]). Renal damage is a major limiting factor for treatment, whereas CIN is a dose-limiting factor for cisplatin, with a reported incidence rate ranging from 20% to 30% [1,2]. If renal damage occurs, stopping the treatment with anticancer drugs or reducing their dosage may be necessary, and cancer progression itself due to the inability to maintain the planned intensity of treatment may affect prognosis [3]. Thus, preventing renal damage is extremely important.

Several strategies have been devised to prevent CIN, such as ensuring sufficient hydration, providing electrolyte supplementation, and optimizing cisplatin administration protocols [4,5,6,7]. Despite advancements in supportive care, CIN continues to be a significant clinical adverse event that can restrict cisplatin treatment. Additionally, hypomagnesemia is a prevalent electrolyte abnormality linked to cisplatin therapy, potentially contributing to CIN development by increasing renal tubular accumulation of cisplatin [6,8]. Hypomagnesemia and hypocalcemia result from the loss of magnesium in the kidneys due to damage to renal tubular cells and calcium-/magnesium-sensitive receptors, renal tubular dysfunction, and magnesium loss from the gastrointestinal tract associated with vomiting and diarrhea caused by cisplatin [9]. Hypomagnesemia has been identified as a risk factor for impaired renal function recovery among patients with severe acute kidney injury (AKI) [10] and has been suggested to cause or worsen renal damage via cisplatin accumulation in the proximal tubules. Magnesium supplementation during cisplatin-based chemotherapy has been shown to correlate with CIN prevention [11,12,13]. Nonetheless, previous studies have not ascertained whether the amount of magnesium supplementation is the same and whether intravenous magnesium supplementation at doses of 8 mEq [14,15,16], 20 mEq [17,18], and 40 mEq [11] exerts a preventive effect against CIN. Additionally, 20 mEq and 8 mEq magnesium supplementation doses for CIN reportedly show no significant difference [19]. Prophylactic magnesium administration may prevent renal damage and does not affect the side effects or therapeutic effects of chemotherapy [20]. However, studies on the effect of renal damage reduction have small sample sizes, making analysis difficult. Furthermore, while several studies have documented the prevention of renal damage through magnesium administration [12,14,15,16,17,18], a prior meta-analysis explored the relationship between magnesium dosage and the prevention of CIN [19]. However, this meta-analysis was limited by the inclusion of a small number of studies and did not thoroughly assess the impact of cisplatin dosage or the dose–response relationship through subgroup analyses and meta-regression. Consequently, the current meta-analysis seeks to provide updated evidence on the preventive effect of magnesium supplementation against CIN and to determine whether the magnesium dose affects its renoprotective efficacy.

## 2. Methods

### 2.1. Protocol

This study was conducted in accordance with the Preferred Reporting Items for Systematic Reviews and Meta-analyses (PRISMA) reporting guidelines [21]. The completed PRISMA 2020 checklist is provided in Supplementary Table S1. As this meta-analysis was performed using the results of previously published studies, the acquisition of ethical approval or informed consent was not required for the present study.

### 2.2. Search Strategy

PubMed, Cochrane Library, Scopus, and Ichushi databases were searched for relevant studies from inception to 31 December 2025. No language restrictions were applied. A manual search based on the references and relevant secondary sources of the studies identified in the database search was conducted using (“cisplatin”) and (“magnesium”) as search terms to provide a comprehensive collection of relevant articles. The search terms are detailed in the Supplementary Materials.

### 2.3. Study Selection Criteria

The eligibility criteria were as follows: (i) studies enrolling adult patients who were diagnosed with solid tumors and underwent cisplatin-based chemotherapy; (ii) studies comparing the effect of magnesium supplementation in intervention versus control groups; and (iii) studies reporting nephrotoxicity-related outcomes. Studies without a comparator group (e.g., case series or single-arm studies) were excluded, whereas both randomized and non-randomized comparative studies were included. Studies were not excluded based on the observation period or publication year.

Two independent reviewers (M.N. and K.J.) screened the titles and abstracts, and eligibility was subsequently assessed based on the full texts. The original authors were not contacted when relevant data were missing. Disagreements between the two reviewers were resolved through discussion; if this failed, a third reviewer acted as an arbiter (T.I.).

### 2.4. Statistical Analysis

All statistical analyses were performed using EZR version 1.29 (Saitama Medical Center, Jichi Medical University, Saitama, Japan). Heterogeneity between studies was examined using the chi-square test and *I*^2^ measure. When substantial heterogeneity (*I*^2^ > 50%) was identified, *p*-values < 0.10 were defined as statistically significant [22]. Fixed- and random-effects models were used for homogeneous and heterogeneous data, respectively. All pooled relative odds ratios (ORs) and 95% confidence intervals (CIs) were calculated using a fixed-effects model with the Mantel–Haenszel method. Meta-regression analysis was conducted to verify the validity of stratification. Magnesium dose was stratified into three strata (namely, <10 mEq, 10 to <20 mEq, and ≥20 mEq); ORs were estimated using a random-effects model for groups with significant heterogeneity and a fixed-effects model for groups with little heterogeneity. Cisplatin dose was also stratified into two groups (namely, ≤60 mg/m^2^ and >60 mg/m^2^), with calculations being performed using a fixed-effects model. Subgroup analyses were performed to assess heterogeneity. Publication bias was assessed using funnel plots and further evaluated quantitatively using Egger’s regression asymmetry test.

### 2.5. Risk-of-Bias Assessment

The risk of bias was independently examined, and the results were cross-checked using the Newcastle–Ottawa Scale (NOS) [23], which is a valid method for evaluating the quality of systematic reviews of observational studies. The NOS consists of eight items across three domains: selection of study participants (4 points), control of confounding factors in the study cohort (1 point), and adjudication of outcome events (3 points). The total score is 8.0 points, with scores of 7.0–8.0 and 4.0–6.0 indicating high and moderate quality, respectively. M.N. and K.J. independently performed the assessment.

## 3. Results

### 3.1. Search Results

**Figure 1:**
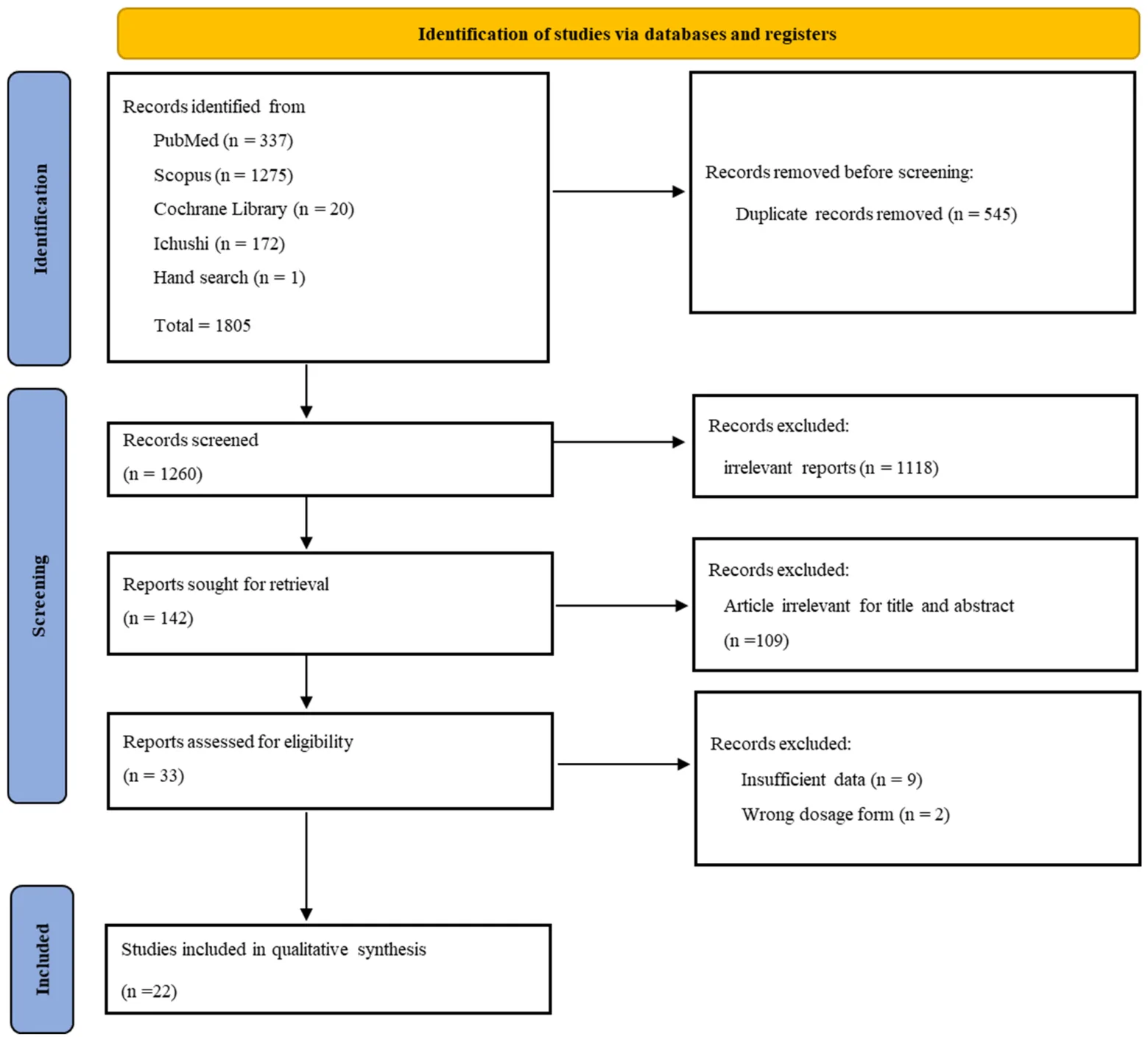
PRISMA flowchart diagram illustrating the study selection process. The systematic process of identifying, screening, qualifying, and including studies in the meta-analysis is shown. A total of 1805 records were identified through database searches (PubMed, Scopus, Cochrane Library, and Ichushi). Following the removal of duplicates (*n* = 545), 1260 records were screened. Ultimately, 22 studies met the inclusion criteria and were included in the meta-analysis.

A flow diagram of the study screening process is shown in Figure 1. A total of 1805 articles were identified through the database search. Following the removal of duplicates, 1214 articles were included; however, 1118 irrelevant articles were excluded. Out of 142 remaining articles, 109 were excluded based on abstract evaluation. Thirty-three remaining articles were reviewed, and 22 studies (17 retrospective cohort studies, three prospective studies, one randomized controlled trial, and one non-randomized intervention trial) were finally included in the meta-analysis. The characteristics of these studies are summarized in Table 1, whereas the NOS evaluation results for the 22 included studies are presented in Table 2. All articles had NOS scores of >6, indicating a low risk of bias.

### 3.2. Renoprotective Effect of Magnesium Administration and Renoprotective Effect by Magnesium Administration Dose

**Figure 2:**
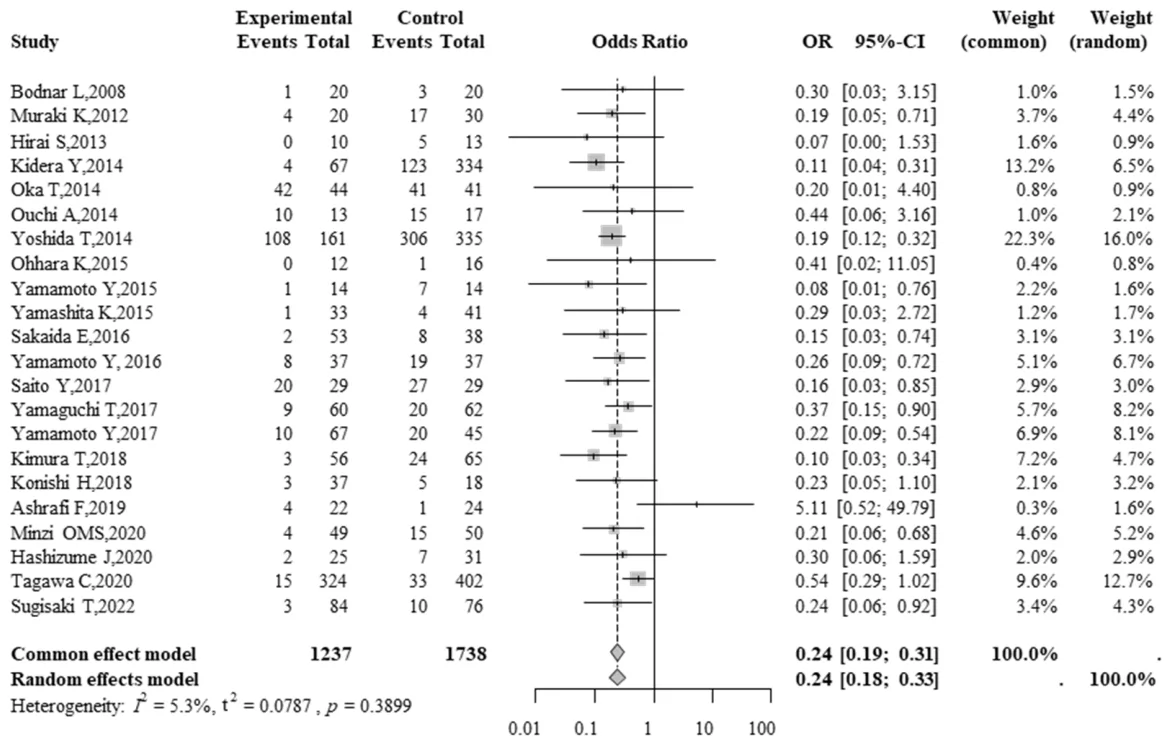
Forest plot showing the incidence of cisplatin-induced renal dysfunction with magnesium administration [11,14,17,20,24,25,26,27,28,29,30,31,32,33,34,35,36,37,38,39,40,41].

**Figure 3:**
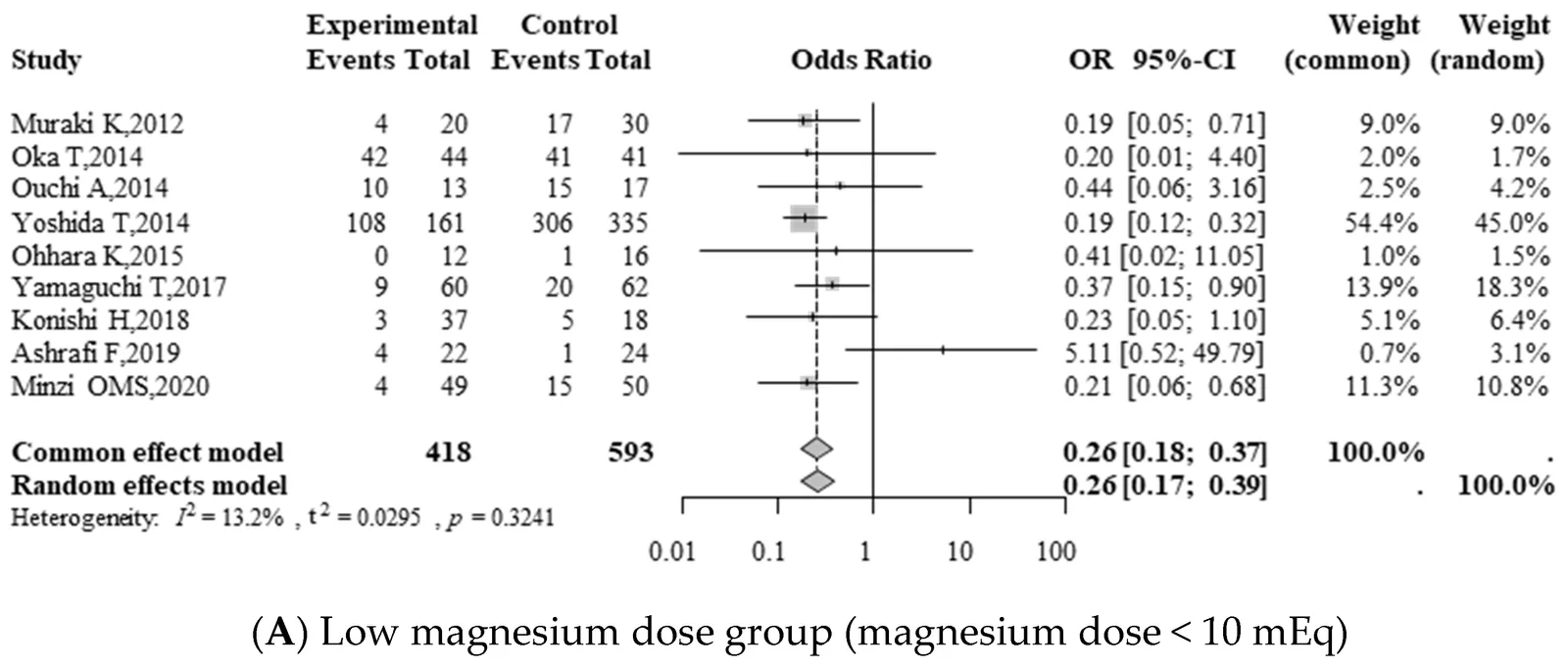

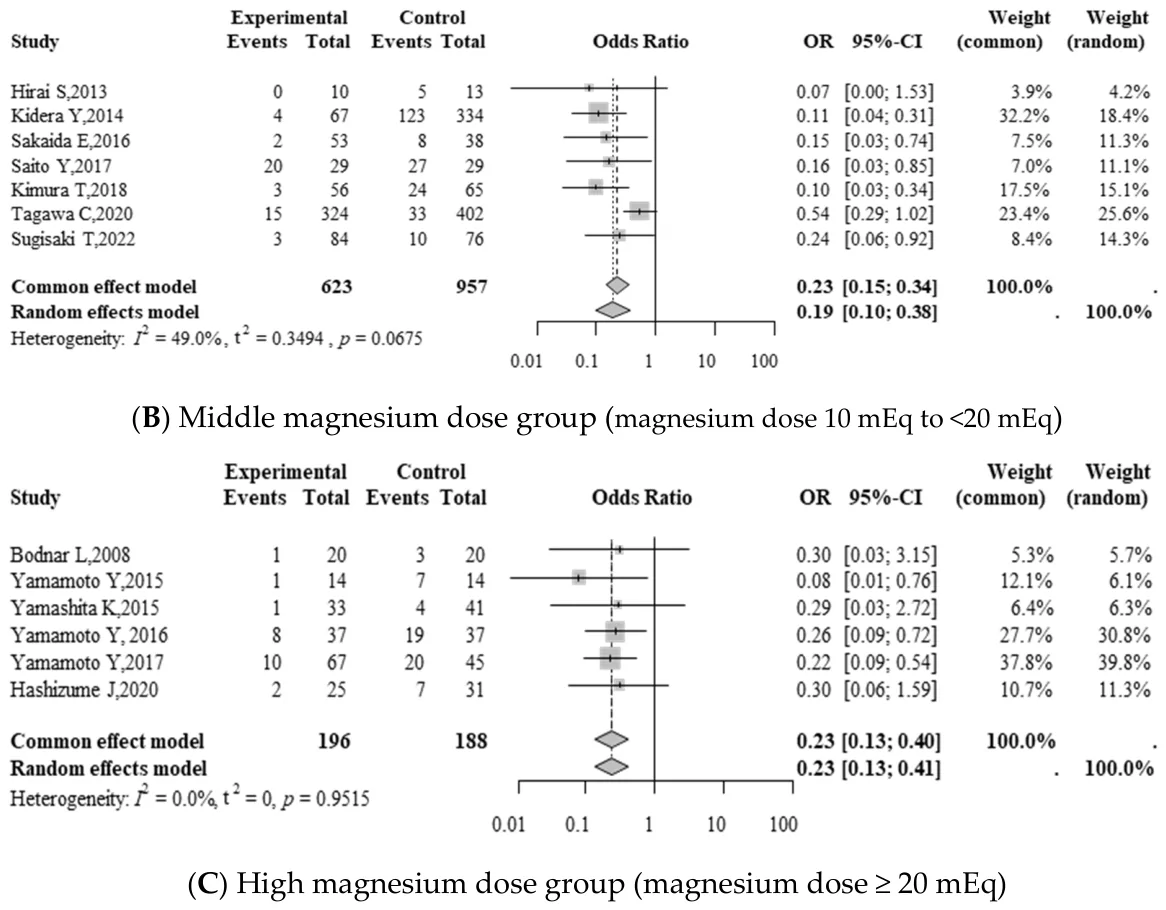
Subgroup analyses. Forest plots showing the incidence of cisplatin-induced renal dysfunction in the (**A**) low magnesium dose group (magnesium dose < 10 mEq), (**B**) middle magnesium dose group (magnesium dose 10 mEq to <20 mEq) and (**C**) high magnesium dose group (magnesium dose ≥ 20 mEq) [11,14,17,20,24,25,26,27,28,29,30,31,32,33,34,35,36,37,38,39,40,41].

The forest plots of the meta-analysis conducted in this study are presented in Figure 2. No heterogeneity was observed (degree of heterogeneity: *I*^2^ = 5.3%, *p* = 0.389), and ORs were calculated using a fixed-effect model. Magnesium dose was categorized into <10 mEq, 10 to <20 mEq, and ≥20 mEq. Subgroup analyses were conducted for each dose category, as shown in Figure 3A–C, with fixed-effects models applied to all analyses.

The pooled analysis indicated that magnesium supplementation significantly decreased the risk of cisplatin-induced nephrotoxicity (OR, 0.24; 95% CI, 0.18–0.33; Figure 2). Subgroup analyses further revealed that magnesium supplementation significantly lowered the risk across all dose categories: <10 mEq (OR, 0.26; 95% CI, 0.18–0.37; Figure 3A), 10 to <20 mEq (OR, 0.23; 95% CI, 0.15–0.34; Figure 3B), and ≥20 mEq (OR, 0.23; 95% CI, 0.13–0.40; Figure 3C).

### 3.3. Meta-Regression Analysis of the Relationship Between Magnesium Dose and Renoprotective Effect

**Figure 4:**
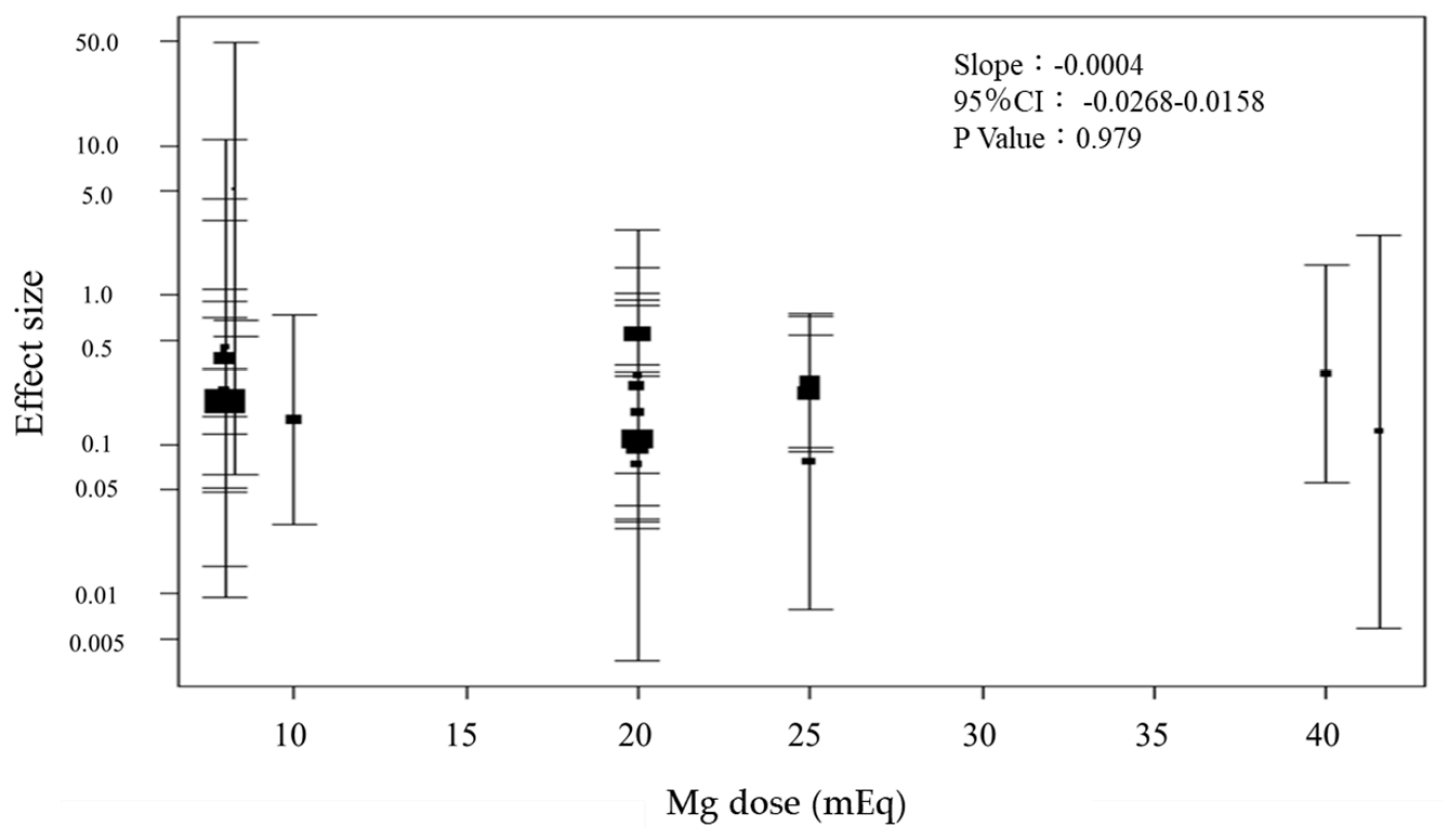
Meta-regression analysis according to magnesium supplementation dose.

A meta-regression analysis was conducted with magnesium dose as a continuous variable to explore the relationship between magnesium dose and renoprotective effect (Figure 4). Such analysis revealed no statistically significant association between magnesium dose and the OR for nephrotoxicity (slope = −0.0004, 95% CI: −0.0268 to 0.0158, *p* = 0.979), indicating that increasing magnesium dose did not significantly alter the protective effect. This result supported the findings of the subgroup analyses and suggested that a lower magnesium dose may be as effective as a higher magnesium dose in preventing CIN.

### 3.4. Renoprotective Effect of Magnesium Administration and Renoprotective Effect by Cisplatin Administration Dose

**Figure 5:**
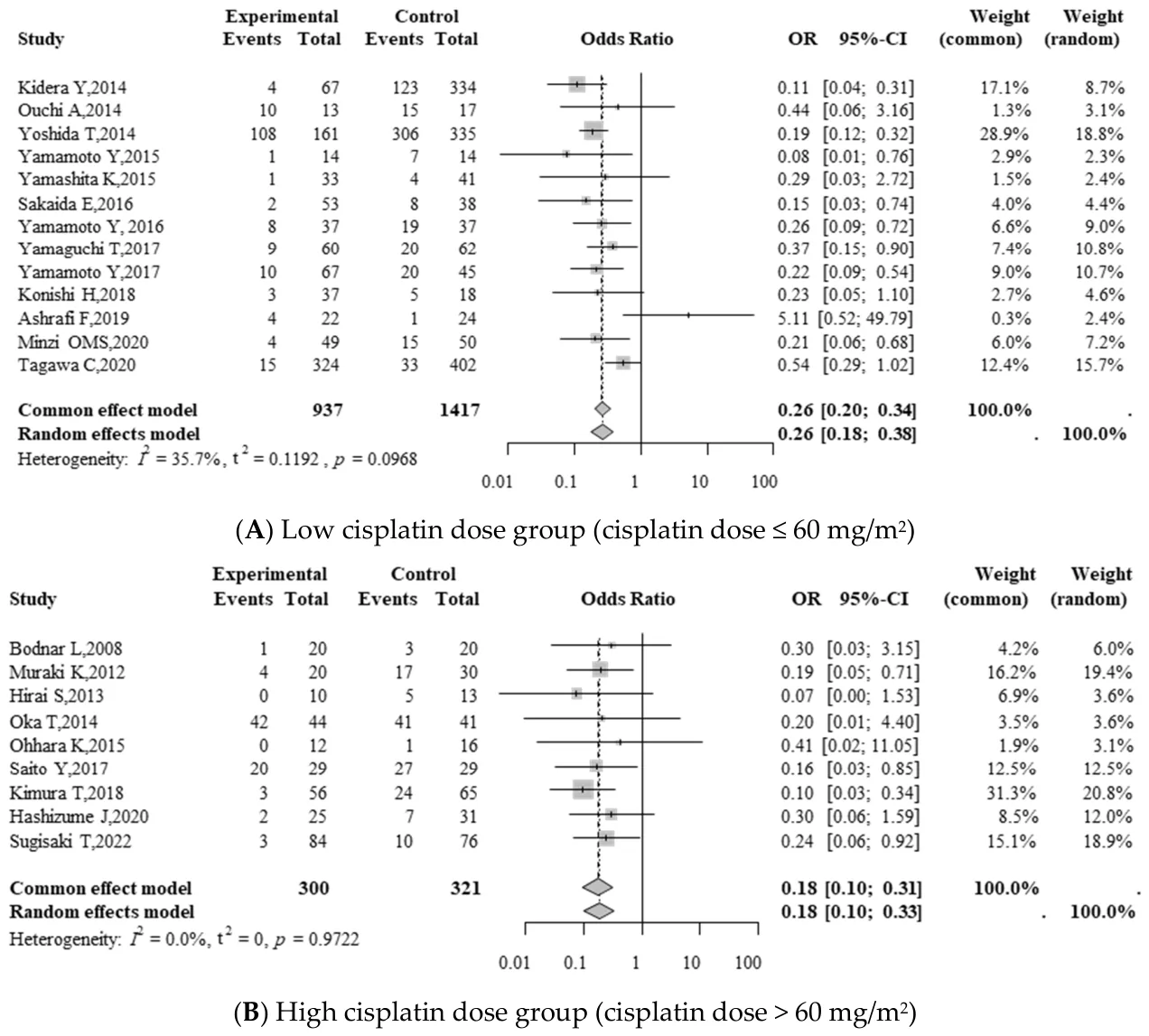
Forest plots of subgroup analyses stratified by cisplatin dose. (**A**) Low-dose group (≤60 mg/m^2^), (**B**) High-dose group (>60 mg/m^2^). [11,14,17,20,24,25,26,27,28,29,30,31,32,33,34,35,36,37,38,39,40,41].

The results of analysis stratified by cisplatin dose are presented in Figure 5A,B. Magnesium supplementation was associated with a significantly reduced risk of cisplatin-induced nephrotoxicity in both the ≤60 mg/m^2^ group (OR, 0.18; 95% CI, 0.10–0.31) and the >60 mg/m^2^ group (OR, 0.26; 95% CI, 0.18–0.38).

### 3.5. Evaluation of the Existence of Publication Bias

**Figure 6:**
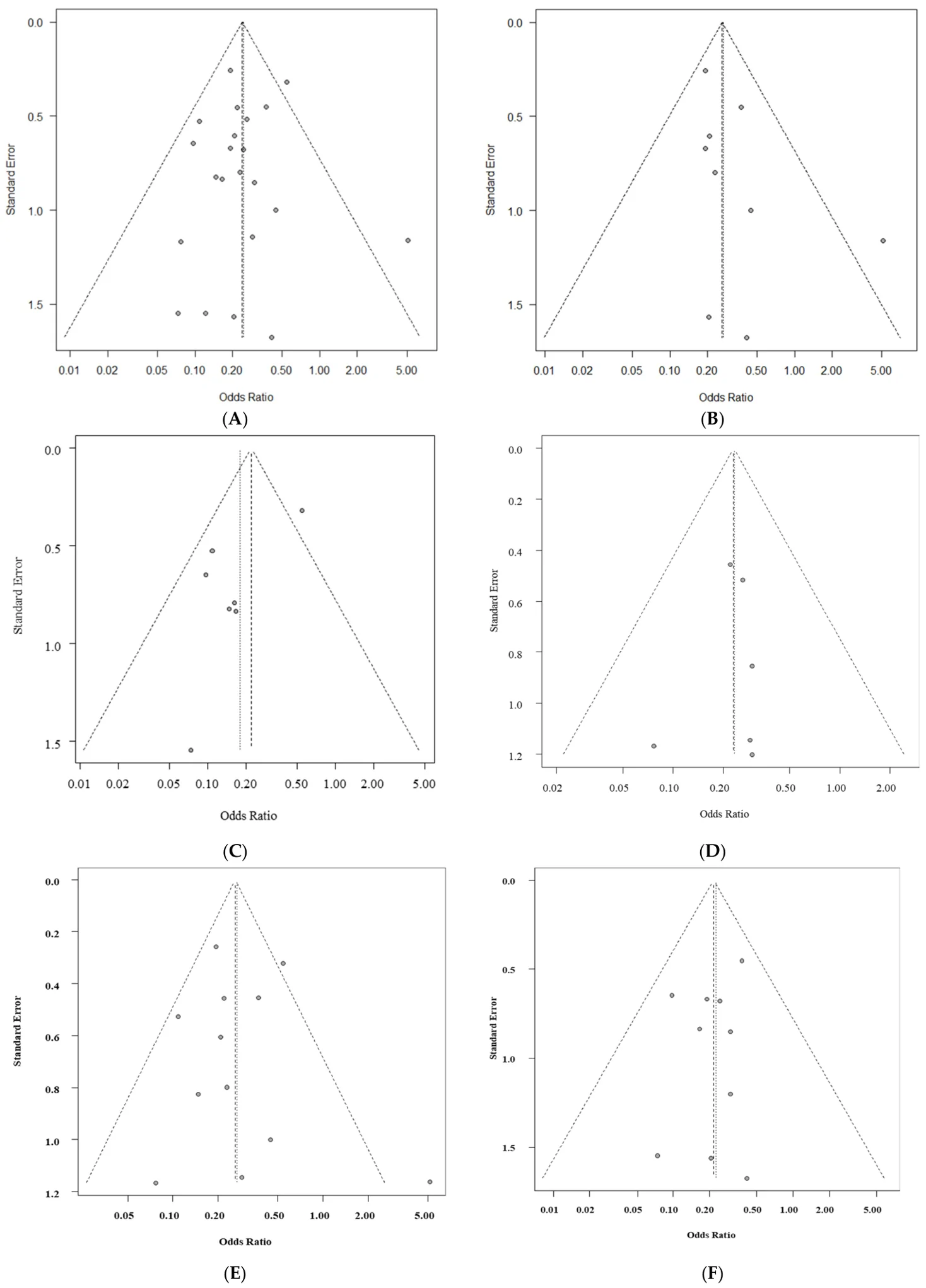
Funnel plots for visual assessment of publication bias. (**A**) Overall analysis. (**B**–**D**) Subgroup analyses according to magnesium supplementation dose (<10 mEq, 10–<20 mEq, and ≥20 mEq, respectively). (**E**,**F**) Subgroup analyses according to cisplatin dose (≤60 mg/m^2^ and >60 mg/m^2^, respectively).

Publication bias was visually assessed using a funnel plot (Figure 6A); no obvious asymmetry was observed. Furthermore, the results of Egger’s regression test, which was performed to statistically examine the influence of small studies, indicated no significant publication bias in the overall analysis (Egger’s test, *p* = 0.38).

The publication bias in the subgroup analyses of magnesium supplementation dose is shown in Figure 6B–D. No significant evidence of bias regarding magnesium supplementation doses < 10 mEq, 10–20 mEq, and ≥20 mEq was found (*p* = 0.41, *p* = 0.0542, and *p* = 0.8904, respectively). Furthermore, the subgroup analyses of cisplatin dose showed no significant evidence of publication bias in the ≤60 mg/m^2^ and >60 mg/m^2^ groups (*p* = 0.4721 and *p* = 0.4766, respectively) (Figure 6E,F).

## 4. Discussion

Magnesium supplementation has been widely used in clinical practice to prevent CIN, and its renoprotective effects have been reported by several previous studies. Recent guidance on cisplatin administration has also emphasized magnesium supplementation as an important component of nephroprotective strategies [42]. Nonetheless, the optimal magnesium dosage remains controversial, and previous investigations into the renoprotective effects of magnesium have often been limited by small sample sizes or single-center designs. To the best of our knowledge, the present study represents one of the most comprehensive meta-analyses on this topic. In particular, the present meta-analysis showed that magnesium supplementation significantly reduced the risk of CIN. Notably, no significant dose–response relationship was observed, suggesting that the protective effect is independent of magnesium dose.

Recently, Gupta et al. reported that prophylactic intravenous magnesium administration was associated with a lower risk of the composite outcome of cisplatin-associated acute kidney injury or death in a large multicenter cohort study [43]. Although their findings support the potential nephroprotective effect of magnesium supplementation, differences in the definitions of cisplatin-induced nephrotoxicity should be considered when comparing their results with those of our meta-analysis.

A well-known side effect of cisplatin is hypomagnesemia, which reportedly occurs in approximately 90% of patients receiving cisplatin [8], along with renal damage. The degree of magnesium depletion has been suggested to depend on the cumulative cisplatin dose [44]. Cisplatin is excreted in the urine via glomerular filtration and tubular secretion, and various transporters mediate the secretion of cisplatin in proximal tubules. In humans, cisplatin enters tubular cells basolaterally via organic cation transporter 2 (OCT2) and accumulates in these cells before being transported to the urine on the apical side via multidrug and toxin extrusion transporters. Some OCT2 inhibitors have been shown to reduce cisplatin-induced cytotoxicity and CIN [45]. Direct damage to the renal tubules by cisplatin often leads to AKI (20–30%) and affects the renal reabsorption capacity, including magnesium reabsorption, resulting in renal magnesium loss and hypomagnesemia (40–100%) [46]. Magnesium loss from the digestive tract associated with vomiting and diarrhea caused by cisplatin administration is also involved. Cisplatin accumulation in the proximal tubules during hypomagnesemia may lead to or worsen renal damage [6,9]. Magnesium supplementation has been reported to improve renal function, indicating that magnesium is an effective protective agent against CIN. The exact mechanism involved in this renal function protection remains poorly understood; nevertheless, it is thought to be related to alterations in renal tubular transport [8,44]. The majority of the included studies involved patients who were treated with high-dose cisplatin (>50 mg/m^2^). Magnesium supplementation for these patients may be beneficial, given the significant decline in the glomerular filtration rate and magnesium concentration [47]. Owing to the reported increased risk of gastrointestinal side effects with oral administration [13] and the small number of reports, the present analysis included only studies that involved intravenous magnesium supplementation. However, no study has directly compared the effects of intravenous magnesium alone and oral magnesium addition on CIN prevention. Additionally, most studies were conducted prior to cisplatin administration, and magnesium sulfate (MgSO_4_) was used in all cases. In most reports, the cisplatin dose analyzed exceeded 60 mg/m^2^. Furthermore, this analysis indicated no significant difference in the inhibitory effect of magnesium supplementation on renal damage due to differences in the cisplatin dose. This is reasonable because cisplatin affects the kidney within 6 h after the start of cisplatin-based chemotherapy and urinary markers of proximal tubular toxicity increase within 3–6 h after chemotherapy [48]. Our analysis included studies in which magnesium was administered before cisplatin administration to prevent CIN and those in which magnesium was administered separately before and after cisplatin administration, and a renoprotective effect was observed. Given that no studies have examined the relationship between blood magnesium levels after magnesium supplementation and CIN prevention, further investigation should be conducted to determine the appropriate amount of magnesium supplementation. Furthermore, a previous meta-analysis of magnesium’s renoprotective effect against CIN reported a correlation between the amount of magnesium administered and the CIN preventive effect [49]; however, this study yielded a new finding: the amount of magnesium administered was not correlated with the CIN preventive effect. We believe that the differences in results are attributable to factors such as differences in the papers used in the analysis and in the models used when synthesizing the papers because of differences in heterogeneity between studies.

Supplementary subgroup analysis revealed that 10–20 mEq magnesium supplementation significantly prevented the development of CIN in the group receiving a cisplatin dose of <60 mg/m^2^, suggesting that CIN may be preventable even with lower magnesium supplementation doses of 10–20 mEq (Supplementary Figures S1 and S2).

This study had some limitations. First, most studies included in this analysis were observational studies with a high risk of bias, which might have influenced the reliability of our findings. Second, there is inherent subjectivity in the design of this study, including the creation of a list of search terms, subsequent data extraction, and final selection of articles. Reviews were conducted by at least two people at each step to eliminate subjectivity. Third, differences in tumor type, hydration method, cisplatin dose, and criteria and evaluation methods for nephrotoxicity among the studies may have influenced the results. Fourth, supportive care strategies, including the use of diuretics such as mannitol and furosemide, varied across the included studies. Because these differences could not be fully adjusted for, they may have influenced the observed relationship between magnesium dose and nephroprotective effects. Finally, the included studies did not benefit from recent advances in medicine. The oldest study included in this analysis was published in 2008. Since then, new antiemetics, such as 5-HT3 and NK-1 inhibitors, have been launched and have become the standard regimen for suppressing vomiting. Appropriate suppression of vomiting must be considered to prevent depletion of intravascular fluid and electrolytes and to reduce the possibility of AKI due to dehydration. These limitations should be considered when interpreting the results of this study.

Although magnesium supplementation has been shown to be beneficial, the optimal magnesium dosage remains unclear. Given the limitations in the number and quality of available studies, further high-quality randomized controlled trials should be conducted to ascertain the optimal magnesium dosage.

## 5. Conclusions

In this analysis, no difference in the preventive effect on CIN was observed, irrespective of whether the magnesium dose was <10 mEq, 10 to <20 mEq, or ≥20 mEq. Thus, magnesium administration is effective for preventing cisplatin-induced nephropathy, regardless of the dose.

## Figures and Tables

**Table 1:** General characteristics of the included studies.

Study ID	Country	Cancer Type	Study Design	Number of Events (I/C)	Number of Patients (I/C)	Cisplatin Dose (mg/m^2^)	Magnesium Dose	Reported Outcome
Bodnar L (2008) [11]	Poland	Ovarian cancer	Randomized controlled trials	1/3	20/20	75 mg/m^2^	MgSO_4_ 5 g (iv) Magnesium subcarbonate supplementation, 500 mg three times daily (oral)	Grade ≥ 1 (creatinine, CTC NCI Grade)
Muraki K (2012) [24]	Japan	Lung cancer	Retrospective study	4/17	20/30	75 mg/m^2^	MgSO_4_ 8 mEq	Grade ≥ 1 (serum creatinine)
Hirai S (2013) [25]	Japan	Esophageal and hypopharyngeal cancer	Retrospective study	0/5	10/13	80 mg/m^2^	MgSO_4_ 20 mEq	Nephrotoxicity was defined as an increase in serum creatinine levels by ≥0.5 mg/dL above the baseline
Kidera Y (2014) [17]	Japan	All types	Retrospective study	4/123	67/334	≥60 mg/m^2^	MgSO_4_ 20 mEq	Grade ≥ 2 (serum creatinine)
Oka T (2014) [26]	Japan	Lung cancer	Retrospective study	42/41	44/41	80 (75–80) mg/m^2^	MgSO_4_ 8 mEq	Grade ≥ 1 (serum creatinine)
Ouchi A (2014) [27]	Japan	Gastric cancer, lung cancer, or urothelial cancer	Retrospective study	10/15	13/17	≥60 mg/m^2^	MgSO_4_ 8 mEq	Grade ≥ 1 (creatinine increase by the Cockcroft–Gault formula)
Yoshida T (2014) [14]	Japan	Thoracic malignancy	Retrospective study	108/306	161/335	≥60 mg/m^2^	MgSO_4_ 8 mEq	Grade ≥ 1 (serum creatinine)
Ohhara K (2015) [28]	Japan	Lung cancer	Retrospective study	0/1	12/16	≥60 (60 or 80) mg/m^2^	MgSO_4_ 8 mEq	Grade ≥ 2 (serum creatinine)
Yamamoto Y (2015) [29]	Japan	Cervical cancer	Prospective, open-label, non-randomized, historically controlled study	1/7	14/14	40 mg/m^2^	Day 1, MgSO_4_ 15 mEq Days 2–3, MgSO_4_ 5 mEq	RIFLE criteria Class R
Yamashita K (2015) [30]	Japan	Lung cancer	Retrospective study	1/4	33/41	≥60 mg/m^2^	MgSO_4_ 20 mEq 26 cases MgSO_4_ 40 mEq 7 cases	Grade ≥ 1 (serum creatinine)
Sakaida E (2016) [31]	Japan	Lung cancer	Retrospective study	2/8	53/38	≥60 mg/m^2^	MgSO_4_ 10 mEq	Grade ≥ 1 (serum creatinine)
Yamamoto Y (2016) [32]	Japan	Gynecological cancer	Prospective, open-label, non-randomized, historically-controlled study	8/19	37/37	≥50 mg/m^2^	Day 1, MgSO_4_ 15 mEq Days 2–3, MgSO_4_ 5 mEq	RIFLE criteria Class R
Saito Y (2017) [20]	Japan	Head and neck cancer	Retrospective study	20/27	29/29	75 mg/m^2^	MgSO_4_ 20 mEq	Grade ≥ 1 (serum creatinine)
Yamaguchi T (2017) [33]	Japan	Lung cancer	Retrospective study	9/20	60/62	≥50 mg/m^2^	MgSO_4_ 8 mEq	Grade ≥ 1 (serum creatinine)
Yamamoto Y (2017) [34]	Japan	Lung cancer	Retrospective study	10/20	67/45	40–60 mg/m^2^	Day 1, MgSO_4_ 15 mEq Days 2–3, MgSO_4_ 5 mEq	RIFLE criteria Class R
Kimura T (2018) [35]	Japan	Head and neck cancer	Retrospective study	3/24	56/65	80 mg/m^2^	MgSO_4_ 20 mEq	Nephrotoxicity was defined as a decrease in creatinine clearance by 20% after two courses
Konishi H (2018) [36]	Japan	Esophageal squamous cell carcinoma	Prospective cohort study	3/5	37/18	≥60 mg/m^2^	MgSO_4_ 8 mEq	Grade ≥ 1 (serum creatinine)
Ashrafi F (2019) [37]	Iran	All types	Retrospective study	4/1	22/24	≥50 mg/m^2^	MgSO_4_ 1 g	Nephrotoxicity was defined as an increase in serum creatinine levels by ≥0.5 mg/dL above the baseline and a decrease in the glomerular filtration rate by ≥50% during each course of chemotherapy.
Minzi OMS (2020) [38]	Tanzania	Cervical, esophageal, or oral cancer or other cancer (*n* = 1)	Non-randomized intervention study	4/15	49/50	≥50 mg/week.	MgSO_4_ 1 g	Nephrotoxicity was defined as an increase in serum creatinine levels >1.5 times the baseline
Hashizume J (2020) [39]	Japan	Head and neck cancer	Retrospective study	2/7	25/32	80 mg/m^2^	Days 1–4, MgSO_4_ 10 mEq	Grade ≥ 1 (serum creatinine)
Tagawa C (2020) [40]	Japan	All types	Retrospective study	15/33	324/402	≥60 mg/m^2^	MgSO_4_ 20 mEq	Grade ≥ 1 (serum creatinine)
Sugisaki T (2022) [41]	Japan	Esophageal cancer	Retrospective study	3/10	84/76	140–160 mg/m^2^	MgSO_4_ 20 mEq	Grade ≥ 2 (eCcr: estimated creatinine clearance)

**Table 2:** NOS evaluation results.

Study	Cohort Selection (A to D)				Comparability	Outcome (F to H)			Total Score
	A	B	C	D	E	F	G	H	
Bodnar L et al. (2008) [11]	1	1	1	1	1	1	1	1	8
Muraki K et al. (2012) [24]	1	1	0	1	1	1	0	1	6
Hirai S et al. (2013) [25]	1	1	0	1	1	1	0	1	6
Kidera Y et al. (2014) [17]	1	1	0	1	1	1	0	1	6
Oka T et al. (2014) [26]	1	1	0	1	1	1	0	1	6
Ouchi A et al. (2014) [27]	1	1	0	1	1	1	1	1	7
Yoshida T et al. (2014) [14]	1	1	0	1	1	1	1	1	7
Ohhara K et al. (2015) [28]	1	1	0	1	1	1	0	1	6
Yamamoto Y et al. (2015) [29]	1	1	1	1	1	1	1	1	8
Yamashita K et al. (2015) [30]	1	1	0	1	1	1	1	1	7
Sakaida E et al. (2016) [31]	1	1	0	1	1	1	1	1	7
Yamamoto Y et al. (2016) [32]	1	1	1	1	1	1	1	1	8
Saito Y et al. (2017) [20]	1	1	0	1	1	1	1	1	7
Yamaguchi T et al. (2017) [33]	1	1	0	1	1	1	1	1	7
Yamamoto Y et al. (2017) [34]	1	1	0	1	1	1	1	1	7
Kimura T et al. (2018) [35]	1	1	0	1	1	1	1	1	7
Konishi H et al. (2018) [36]	1	1	0	1	1	1	0	1	6
Ashrafi F et al. (2019) [37]	1	1	0	0	1	1	1	1	6
Minzi OMS et al. (2020) [38]	1	1	1	1	1	1	0	1	7
Hashizume J et al. (2020) [39]	1	1	0	1	1	1	0	1	6
Tagawa C et al. (2020) [40]	1	1	0	1	1	1	0	1	6
Sugisaki T et al. (2022) [41]	1	1	0	1	1	1	1	1	7

A = representativeness of the exposed cohort, B = representativeness of the non-exposed cohort, C = ascertainment of exposure, D = outcome of interest was not present at the start of the study, E = comparability of exposed and non-exposed cohorts, F = quality of outcome assessment, G = follow-up was long enough for outcomes to occur, H = adequacy of follow-up.

## Data Availability

The data used in this study are derived from publicly available studies cited in Table 1 of the paper. Extracted data supporting the findings of this study are available from the corresponding author upon reasonable request.

## Supplementary Materials

The following supporting information can be downloaded at: https://www.mdpi.com/article/10.3390/jcm15166278/s1. Figure S1: Forest plot of a supplementary subgroup analysis (cisplatin dose <60 mg/m^2^ and magnesium supplementation 10–20 mEq); Figure S2: Funnel plot for supplementary subgroup analysis (cisplatin dose <60 mg/m^2^ and magnesium supplementation 10–20 mEq); Table S1: PRISMA 2020 Checklist.

## Abbreviations

The following abbreviations are used in this manuscript:

AKI	Acute kidney injury
CI	Confidence interval
CIN	Cisplatin-induced nephrotoxicity
NOS	Newcastle–Ottawa Scale
OCT2	Organic cation transporter 2
OR	Odds ratio
PRISMA	Preferred Reporting Items for Systematic Reviews and Meta-Analyses
